# Developmental trajectories of expert perception processing of Chinese characters in primary school children

**DOI:** 10.3389/fpsyg.2022.932666

**Published:** 2022-08-01

**Authors:** Yini Sun, Jianping Wang, Qing Ye, Baiwei Liu, Ping Zhong, Chenglin Li, Xiaohua Cao

**Affiliations:** ^1^Department of Psychology, Zhejiang Normal University, Jinhua, China; ^2^Department of Biological Psychology and Cognitive Neurosciences, Institute of Psychology, Friedrich-Schiller-Universität Jena, Jena, Germany; ^3^Key Laboratory of Intelligent Education Technology and Application of Zhejiang Province, Zhejiang Normal University, Jinhua, China

**Keywords:** Chinese character, inversion effect, left-side bias effect, development, cognition

## Abstract

Previous studies have demonstrated that inversion effect and left-side bias are stable expertise markers in Chinese character processing among adults. However, it is less clear how these markers develop early on (i.e., among primary school students). Therefore, this study aimed to investigate the development of the two markers by comparing primary school-aged students of three age groups (Grade 1, Grade 3, and Grade 5) and adults in tests of inversion effect (Experiment 1) and left-sided bias effect (Experiment 2). The results replicated that both effects during Chinese character processing were present among adults. However, more importantly, the effects were different among primary school-aged students in different grades: the inversion effect was found as early as in Grade 1, but the left-side bias effect did not emerge in Grade 1 and as approximated that of adults until Grade 3. The study suggested a potential dissociation in developing different aspects of expertise during Chinese character processing in early childhood.

## Introduction

Perception processing is an important step in object recognition. Due to continuous exposure throughout our lives, most people are exceptionally proficient at recognizing visual objects; for example, faces and words. Most adults are experts at face and word recognition. Several behavioral markers in expert perception processing have been identified, such as inversion effect and left-side bias (Yovel, 2016). The inversion effect is commonly considered as evidence of perceptual expertise processing (de Heering et al., 2007, 2012; Robbins and Mckone, 2007; Macchi et al., 2009; Picozzi et al., 2009; Rezlescu et al., 2017). The inversion effect is referred to as a disproportionate impairment of face or word recognition induced by inversion, as compared to object recognition induced by inversion (Yin, 1969; Tien et al., 2008). Left-side bias is another behavioral marker that is consistently reported in expert perception. The left-side bias effect refers to the effect usually associated with chimeric faces; specifically, chimeric faces composed of two left-halves are often judged to be more similar to the original face than chimeric faces composed of two right-halves (Brady et al., 2005). Previous studies that investigated expert processing have focused on face or non-face (e.g., car, bird, and greeble) processing and have found existing stable inversion effect and left-side bias effect in face processing by adults. To reach this expert stage of face processing takes many years. For example, the face inversion effect as a marker of perceptual expertise is mature in young children (at about 6 years), but the expert ability measure by composite paradigm continues to develop into adolescence (Maurer et al., 2002; de Heering et al., 2012).

It is significant to note that due to extensive exposure, words are one of the most familiar visual stimuli, and with the popularity of mass education, they have become one of the few visual stimuli that we formally study. The behavioral and neural evidence of the inversion effect (Kao et al., 2010; Zhao et al., 2010; Wang et al., 2011) and the left-side bias effect (Tso et al., 2014; Li and Cao, 2017) were observed in Chinese character processing. The evidence noted above suggests that there are stable inversion effects and left-side bias in Chinese character processing by adults. What is not yet known, however, is how these markers of expert perception processing of Chinese characters develop in primary school student.

This study aimed to investigate the development of the inversion effect and the left-side bias effect in Chinese character processing by conducting two experiments among primary school students. Experiment 1 investigated the developmental trend of the inversion effect in Chinese character processing by primary school students, and Experiment 2 tested the participants for evidence of left-side bias development in Chinese character processing. The recent ERP studies have demonstrated that the neural mechanisms underlying the expert perception processing of Chinese characters have emerged in second-grade primary school students, who had about 1-year experience of learning Chinese characters, and that by the sixth grade, that is, after about 5-year experience of learning Chinese characters, children displayed the levels of performance similar to adults (Cao et al., 2011; Cao and Zhang, 2011). Therefore, we expected that the developmental trajectory of behavioral expertise markers (i.e., inversion and left-side bias effects) in Chinese character processing may also be observed in primary school students. Specifically, the inversion effect was related to holistic or (more general) configural processing in upright faces, which is impaired in inverted faces (Sagiv and Bentin, 2001; Peterson and Rhodes, 2003), whereas the left-side bias effect was attributed to the processing by the dominant hemisphere (Yovel et al., 2008; Proietti et al., 2015) and/or reading habits (Megreya and Havard, 2011). Therefore, we expected that the inversion effects and the left-side bias may show different developmental characteristic or trajectories in primary school students.

## Experiment 1: The development of the inversion effect in Chinese character processing

### Methods

#### Participants

A total of 60 Chinese children were recruited from a local primary school in Jinhua, China, with 20 each of Grade 1 (6–7 years, *M* = 6.80, *SD* = 0.41 years, 10 females), Grade 3 (8–9 years, *M* = 8.45, *SD* = 0.51 years, 10 females), and Grade 5 (10–12 years, *M* = 10.35, *SD* = 0.49 years, 10 females). The sample size rationale was based on the power of 0.80 and η*_*p*_*^2^ of 0.14 (Wang et al., 2011) using G power 3.1, which generated a need of at least 15 participants per group. Informed consent was obtained from all parents. To measure participants’ prior linguistic knowledge, all participants were tested on a Standardized Chinese Characters Recognition Test (Wang and Tao, 1996). The mean numbers of receptive Chinese characters (the number of print Chinese characters that one could recognize) in each grade were 674.09 for Grade 1 (*SD* = 219.05), 2289.53 for Grade 3 (*SD* = 247.68), and 3123.21 for Grade 5 (*SD* = 160.56). The tests were carried out in July, at which point the students in Grade 1 had received approximately 10 months of formal educational training since September in the previous year. A total of 20 Chinese college students (20–24 years, 10 women) were recruited from Zhejiang Normal University. All participants had normal or corrected-to-normal vision. The study was approved by the ethical committee of Zhejiang Normal University and was carried out in accordance with the approved guidelines. Written consent was obtained from all participants prior to the study. None of the participants were informed of or familiar with the study goal prior to their participation.

#### Materials

**FIGURE 1:**
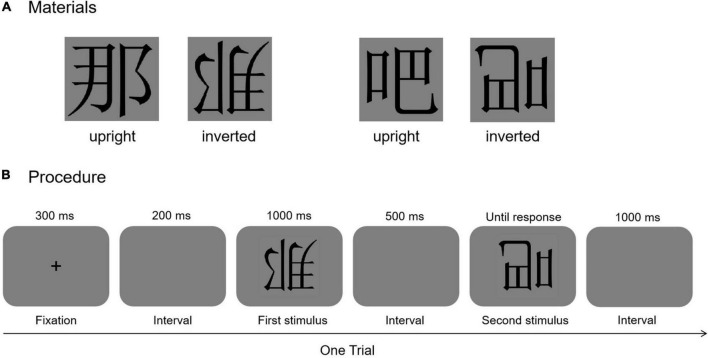
**(A)** Examples for upright and inverted Chinese characters. **(B)** The trial structure of the inversion task.

A total of 20 Chinese characters were chosen from the Beijing Language Institute (1986) (Figure 1). All were high-frequency characters (more than 400 occurrences per 1.31 million, *M* = 0.00277476, *SD* = 0.00208377, ranging from 0.0003169 to 0.009784) with left-right structure that had previously been taught to the Grade 1 students in primary school to ensure that each character was familiar to all primary school students. The number of strokes varied from 6 to 14 (*M* = 6.04, *SD* = 0.77). The characters were presented in black, Song font. Each Chinese character spanned 4.5 × 4° of visual angle at a viewing distance of 60 cm. The 20 Chinese characters were presented in both upright and inverted orientations.

#### Procedure

The procedure is shown in Figure 1. Participants were required to sit at a distance of 60 cm from a 17″ cathode ray tube monitor (CRT; 1,024 × 768 pixel resolution) on which stimuli were presented on a dark gray background. E-Prime 2.0 software (Psychology Software Tools, Pittsburgh, PA, United States) was used to present the stimuli and collect the behavioral responses. Participants were asked to finish a same-different matching task. The Chinese characters were shown in the center of the screen. In each trial, participants viewed a fixation cross-centered on the screen for 300 ms and then the dark gray background for 200 ms, followed by the first stimulus for 1,000 ms, and then the dark gray background for another 500 ms. The second stimulus was then presented until participants pressed a key in response. Participants were asked to match the identity of the two stimuli as accurately and quickly as possible. Half of the participants were asked to press “L” if the two stimuli were identical and “A” if they were different; the key pressing requirements were reversed for the other half of participants. A 1,000 ms presentation of the dark gray background followed each response, following which the next trial began. The paired Chinese characters were presented pseudorandomly in the same orientation.

A total of 160 trials (80 upright and 80 inverted trials) were presented randomly in two blocks.

### Results

**FIGURE 2:**
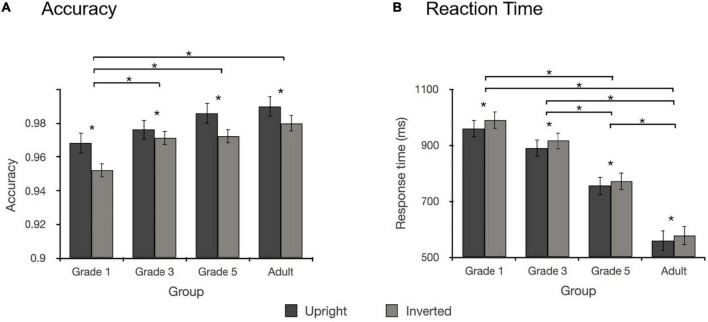
**(A)** The impact of the inversion effect on accuracy in Experiment 1. **(B)** The impact of the inversion effect on RT in Experiment 1. Error bars represent the standard error of the mean. *p < 0.05.

A repeated measures analysis of variance (ANOVA) of the accuracies and response times (RT) was conducted for stimulus orientation (upright, inverted) and participant type (Grade 1, Grade 3, Grade 5, and adult) (Table 1 and Figure 2).

**TABLE 1:** The accuracy (± SD) and reaction time (± SD) in Experiment 1.

	Accuracy		RT (ms)	
		
	Upright	Inverted	Upright	Inverted
Grade 1	0.97 ± 0.26	0.95 ± 0.30	960 ± 134	990 ± 145
Grade 3	0.98 ± 0.17	0.97 ± 0.22	890 ± 144	916 ± 144
Grade 5	0.99 ± 0.12	0.97 ± 0.27	756 ± 159	773 ± 165
Adult	0.99 ± 0.17	0.98 ± 0.18	540 ± 85	562 ± 86

The results for accuracy revealed a significant main effect of stimulus orientation [*F*_(1, 76)_ = 26.024, *p* < 0.001, η*_*p*_*^2^ = 0.255]; namely, the accuracy of Chinese character processing in upright condition was significantly higher than that of Chinese character processing in inverted condition. Moreover, a significant main effect of participant type was found [*F*_(3, 76)_ = 5.581, *p* = 0.002, η*_*p*_*^2^ = 0.181]. *Post-hoc t*-test analysis with an adjusted level of significance using Bonferroni correction (*p* = 0.05/6 = 0.0083) showed that the accuracy in Grade 1 was similar to that in Grade 3 [*t*_(38)_ = 1.897, *p* = 0.065], that the accuracy in Grade 1 was marginally significantly lower than that in Grade 5 [*t*_(38)_ = 2.717, *p* = 0.010, Cohen’s *d* = 0.88], and that the accuracy in Grade 1 was significantly lower than that in adults [*t*_(38)_ = 3.798, *p* = 0.001, Cohen’s *d* = 0.97]. Moreover, the accuracy in Grade 5 was similar to that in the adult group. No significant interaction was found between stimulus orientation and participant type [*F*_(3,76)_ = 0.936, *p* = 0.428, η*_*p*_*^2^ = 0.036].

The results of the RT analysis revealed a significant main effect of stimulus orientation [*F*_(1, 76)_ = 29.387, *p* < 0.001, η*_*p*_*^2^ = 0.279], which demonstrated that the RT for Chinese character processing in upright condition was faster than that for Chinese character processing in inverted condition. A significant main effect of participant type was found [*F*_(3, 76)_ = 38.357, *p* < 0.001, η*_*p*_*^2^ = 0.602]. *Post-hoc t*-test analysis with an adjusted level of significance (*p* = 0.0083) revealed that the RT in each participant type was significantly different from the other types [Grade 1 vs. Grade 5: *t*_(38)_ = 4.444, *p* < 0.001, Cohen’s *d* = 1.44; Grade 1 vs. adult: *t*_(38)_ = 11.683, *p* < 0.001, Cohen’s *d* = 3.70; Grade 3 vs. adult: *t*_(38)_ = 9.470, *p* < 0.001, Cohen’s *d* = 2.99; Grade 5 vs. adult: *t*_(38)_ = 5.242, *p* < 0.001, Cohen’s *d* = 1.66; Grade 3 vs. Grade 5: *t*_(38)_ = 2.884, *p* = 0.006, Cohen’s *d* = 0.94], except between Grade 1 and Grade 3 [Grade 1 vs. Grade 3: *t*_(38)_ = 1.620, *p* = 0.113]. No significant interaction was found between stimulus orientation and participant type [*F*_(3,76)_ = 0.403, *p* = 0.751, η*_*p*_*^2^ = 0.016].

The correlation analysis was conducted between the number of Chinese character recognition and the performance of Chinese character processing in upright and inverted conditions, respectively. The correlation analysis in accuracy showed that the number of Chinese character recognition was significantly positively correlated with the accuracy of Chinese character processing in both upright (*r* = 0.361, *p* = 0.007) and inverted condition (*r* = 0.336, *p* = 0.013), which indicated that the better Chinese character literacy, the better the performance of Chinese character processing in both upright and inverted conditions. In RT, the correlation analysis showed that the number of Chinese character recognition was significantly negatively correlated with the RT of Chinese characters processing in both upright (*r* = −0.457, *p* = 0.001) and inverted condition (*r* = −0.462, *p* < 0.001), which indicates that the better Chinese literacy, the faster the Chinese character processing.

## Experiment 2: The development of the left-side bias effect in Chinese character processing

### Methods

#### Participants

A total of 60 Chinese children from a local primary school in Jinhua, China, took part in the experiment. There were three groups of 20 participants each: Grade 1 (6–7 years, *M* = 6.50, *SD* = 0.51 years, 10 females), Grade 3 (8–9 years, *M* = 8.40, *SD* = 0.60 years, 10 females), and Grade 5 (10–11 years, *M* = 10.45, *SD* = 0.51 years, 10 females). The children in Experiment 2 were not recruited from the same school as Experiment 1, because of some difficulties during the data collection (e.g., we should have finished within 1 month) if we performed Experiments 1 and 2 in only one primary school. The sample size rationale was based on the power of 0.80 and η*_*p*_*^2^ of 0.145 (Tso et al., 2020) using G power 3.1, which generated a need of at least 18 participants per group. The informed consent was obtained from all parents. To measure participants’ prior linguistic knowledge, all participants were tested on a Standardized Chinese Characters Recognition Test (Wang and Tao, 1996). The mean numbers of receptive Chinese characters (the number of print Chinese characters that one could recognize) in each grade were 689.62 for Grade 1 (*SD* = 166.98), 2394.87 for Grade 3 (*SD* = 190.90), and 3148.78 for Grade 5 (*SD* = 143.02). A total of 20 Chinese college students (19–24 years, 10 women) were recruited from Zhejiang Normal University, and informed consent was obtained from all participants. The other selection criteria were the same as that in Experiment 1. None of the Experiment 2 participants participated in Experiment 1, and none were familiar with the study goal prior to participating.

#### Materials

**FIGURE 3:**
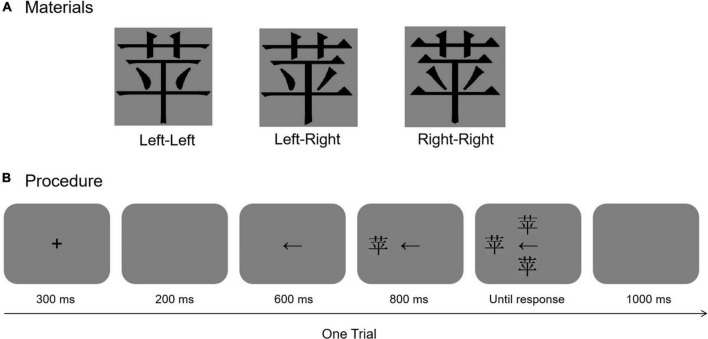
**(A)** Example of chimeric task for Chinese characters. “Left-Left” represents a left chimeric Chinese character image; “Left-Right” represents an original Chinese character image, and “Right-Right” represents a right chimeric Chinese character image. **(B)** Trial structure of the left-side bias task.

A total of 35 mirror-symmetric Chinese characters were chosen from the Beijing Language Institute (1986) (Figure 3). All were high-frequency characters (more than 50 occurrences per 1.31 million, *M* = 0.0007614, *SD* = 0.00153959, ranging from 0.0000437 to 0.0081715) and had previously been taught to the Grade 1 students in the local primary school. The number of strokes in the Chinese characters varied from 6 to 14 (*M* = 6.95, *SD* = 1.64). The Chinese characters were written in black and presented in a widely used standard typeface (Song font). To investigate left-side bias in Chinese character processing, we adapted the method from Li and Cao (2017). We bisected each Chinese character into two halves (left and right) along the vertical midline. The left chimeric character was created from left half of the character with its mirror image, and the right chimeric character was created from right half of the same character with its mirror image. All character images spanned 6° of the visual angles in both width and height from a viewing distance of approximately 60 cm.

#### Procedure

The participants were required to sit at a distance of 60 cm from a 17′′ CRT (1024 × 768 pixel resolution) on which the stimuli were presented on a dark gray background. E-Prime 2.0 software (Psychology Software Tools, Pittsburgh, PA, United States) was used to present the stimuli and collect the behavioral responses. To investigate left-side bias in Chinese character processing, we adapted a procedure similar to that by Hsiao and Cottrell (2009). The procedure is shown in Figure 3. The chimeric Chinese character task consisted of four blocks. The block orders were presented randomly for each participant. The experiment contained 140 trials that were allocated pseudorandomly to four blocks. There were four types [the original Chinese character’s location (left side, right side) × the left chimeric Chinese character’s location (upper side, lower side)], with 35 trials for each type; each original Chinese character appeared once in each block. Each trial started with a central fixation 300 ms in duration, followed by a blank screen for 200 ms. After the blank screen, an arrow pointed randomly to the left or right for 600 ms, and then, the original/reference Chinese character was presented on either the left side or right side of the screen at a visual angle of 7.5° away from the center of the screen for 800 ms. The original Chinese character and its left and right chimeric characters were then presented simultaneously, with one above and the other below the arrow, until the participant responded. There was an approximately 6° visual angle between the two chimeric Chinese characters. Each participant was asked to choose the chimeric Chinese character that was more similar to the original Chinese character. After the response, a dark gray blank screen was presented for 1,000 ms and then the next trial began. Participants were asked to press “T” with the left index finger or “U” with the right index finger if they thought that the upper chimeric Chinese character was more similar to the original Chinese character than the lower chimeric Chinese character and were asked to press “N” with the right index finger or “V” with the left index finger if they thought that the lower chimeric Chinese character was more similar to the original Chinese character than the upper Chinese character. The key assignment (i.e., the instruction to use T/N or U/V) was counterbalanced across participants.

#### Design and data analysis

The independent variable was the participant type (Grade 1, Grade 3, Grade 5, and adult). The dependent variable was the participant’s preference for the left chimeric Chinese character. The preference for the left chimeric Chinese character was calculated as the number of trials in which the participant chose the left chimeric Chinese character divided by the total number of trials (Li and Cao, 2017). Left-side bias was defined as a significantly higher preference for the left chimeric Chinese character than the non-biased threshold of 0.5.

### Results

**FIGURE 4:**
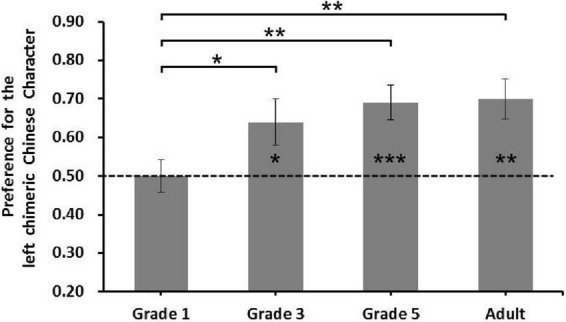
The preference for the left chimeric Chinese character in Experiment 2. Error bars represent the standard error of the mean. *p < 0.05, **p < 0.01, and ***p < 0.001.

The results are shown in Figure 4. One-sample *t*-tests comparing the preference for the left chimeric Chinese character in each grade to the no-bias threshold of 0.5 revealed a reliable left-side bias in Chinese character processing for Grade 3 [*M* = 0.643 ± 0.265, *t*_(19)_ = 2.414, *p* = 0.026, Cohen’s *d* = 0.54], Grade 5 [*M* = 0.685 ± 0.21, *t*_(19)_ = 3.949, *p* = 0.001, Cohen’s *d* = 0.88], and adults [*M* = 0.70 ± 0.18, *t*_(19)_ = 4.776, *p* < 0.001, Cohen’s *d* = 1.11], but not for Grade 1 [*M* = 0.498 ± 0.19, *t*_(19)_ = 0.034, *p* = 0.973]. To examine the development of the left-side bias effect in Chinese character processing, a one-way ANOVA for the preference of the left chimeric Chinese character was conducted for participant type (Grade 1, Grade 3, Grade 5, and adult). The results revealed a significant main effect of participant type [*F*_(3, 76)_ = 3.627, *p* = 0.017, η*_*p*_*^2^ = 0.125]. *Post-hoc t*-test analysis with an adjusted significance level using Bonferroni correction (*p* = 0.05/6 = 0.0083) showed that the preference for the left chimeric Chinese character in Grade 1 was significantly lower than that in Grade 5 [*t*_(38)_ = 2.919, *p* = 0.006, Cohen’s *d* = 0.93] and adults [*t*_(38)_ = 3.399, *p* = 0.002, Cohen’s *d* = 1.09]; the preference in Grade 1 was similar to that in Grade 3 [*t*_(38)_ = 1.988, *p* = 0.054 > 0.0083]. Moreover, there were no significant differences among the other grades; namely, the left-side bias for Chinese character processing in Grade 3 was similar to that in Grade 5 [*t*_(38)_ = 0.551, *p* = 0.585], and among adults [*t*_(38)_ = 0.794, *p* = 0.432]; the left-side bias for Chinese character processing in Grade 5 was similar to that of adults [*t*_(38)_ = 0.244, *p* = 0.808].

The correlation analysis was conducted between the number of Chinese character recognition and the left-side bias effect in Chinese character processing. The results showed that the number of Chinese character recognition was significantly positively correlated with the left-side bias effect (*r* = 0.366, *p* = 0.004), which indicated that the more the number of Chinese character recognition, the stronger the left-side bias in Chinese characters processing.

## Discussion

This study is the first to provide behavioral evidence of the developmental trajectory of Chinese character expert processing in normal primary school students. The results were consistent with previous studies in confirming that both the inversion effect (Kao et al., 2010; Zhao et al., 2010; Wang et al., 2011) and the left-side bias effect (Hsiao and Cottrell, 2009; Tso et al., 2014, 2020; Chung et al., 2017; Li and Cao, 2017) are observed in adults when processing Chinese characters. More importantly, the results demonstrated that the inversion effect in Chinese character processing emerged some times before the end of first grade (in first-grade students with about 10-month experience with Chinese characters as a part of normal primary school education). The left-side bias effect was not observed at all among the first-grade students, but by the third grade, it was already at the adult-like level. The results demonstrated that there is a clear difference between the developmental trajectories of the inversion effect and the left-side bias effect in Chinese character processing in primary school students.

### The developmental performance of the inversion effect in Chinese character processing

This inversion effect was first observed in a study of face processing by Yin (1969). The effect was later replicated with other visual objects of expertise, such as Chinese characters (Kao et al., 2010; Zhao et al., 2010). The face/Chinese character inversion effect describes the observation that both faces and Chinese characters are much more difficult to recognize upside down than other kinds of objects (e.g., Yin, 1969; Kao et al., 2010). The inversion effect reveals the spatial configuration processing involved in a visual analysis of those objects. In the inverted images, the relative spatial relations among image elements are turned upside down, and consequently, this slows down the response of the mechanism that is specialized in the spatial configurations of elements in an image (Kao et al., 2010).

The inversion paradigm is easier for children than other paradigms (e.g., composite paradigm) when use Chinese characters as stimuli; and the composite paradigm for Chinese characters was more influenced by writing experiences/performance (Tso et al., 2014, 2021, 2022). Consequently, in the present experiments, we choose the inversion effect as a measurable index of perceptual expertise. The results of Experiment 1 revealed that the inversion effect of Chinese character processing can be found in first-grade primary school students. This indicated that the inversion effect emerges some times before the end of first grade (within about 1 year of learning Chinese as a part of the school curriculum). This means that the children with about 1 year of experience with Chinese characters have a sensitivity to the expert perception processing that is necessary for comprehending Chinese characters. Moreover, the accuracy in the Grade 1 group was significantly lower than that in both the Grade 5 group and the adult group. This indicates that some (or partial) abilities for processing Chinese characters (i.e., expert perception processing as evidenced from inversion effect) may develop with age. The accuracy in the Grade 5 group was similar to that of adults, which indicated that with about 5-year experience of learning Chinese characters, children reach an adult-like performance in expert perception processing for Chinese characters. Also, the reaction time in Experiment 1 suggested that the speed of processing Chinese characters increases with age. Such a performance increase with age in expert perception processing for Chinese characters, however, may be explained by two factors. First, reading Chinese characters may increase due to a practice effect in an expert perception manner. That is, reading Chinese characters for years facilitates faster reading of Chinese characters. Second, such increase in expert perception processing may also be concurrent with children’ development in general cognitive abilities with age, such as attention, working memory, and inhibitory control (Plude et al., 1994; Schroeter et al., 2004; Crookes and Mckone, 2009; McKone et al., 2012). That is, the enhanced processing speed may be the result Of maturation in task-related cognitive skills, e.g., attention, instead of the practice in reading Chinese characters *per se*. Although this study measured children’ receptive inventory of Chinese characters as a rough indication of “practice effect” and a positive correlation between the children’s receptive inventory and their performance (consistent with Li et al., 2006), it needs caution to explain our results about the development of the inversion effect for Chinese character processing in primary school children, as the interactions between orientation and participant type were not significant in accuracy and RT. The current results lack the power to make a cause-and-effect conclusion that the increase in the perception processing over age results from an expert perception practice effect. Admittedly, teasing apart the two possible reasons for an increase in children’ expert perception processing goes beyond the scope of this study (i.e., expert perception effect of practicing with Chinese characters vs. general effect of a boost in cognitive skills). However, future studies may also measure participants’ individual differences in reading-related cognitive skills (e.g., attention, working memory, and inhibitory control) during children’s Chinese character processing. Most importantly, this study showed that the inversion effect emerged in Grade 1, but there was no significant interaction between orientation and participant type. Consequently, understanding the development trajectories of the inversion effect from its emergence to the adult-like level of Chinese character processing would require a fine-grained investigation on children during the first year of regular learning in a longitudinal study.

Moreover, our study expanded on the evidence from previous studies as it observed the inversion effect in both accuracy and response time analysis (Kao et al., 2010; Wang et al., 2011). Many previous studies have only found the inversion effect from either reaction time or accuracy analysis (Kao et al., 2010; Wang et al., 2011). For example, Kao et al. (2010) found that when the stimuli were presented in both parafoveal and peripheral conditions, the proportional correct response for upright Chinese characters was higher than that for inverted Chinese characters. Wang et al. (2011) demonstrated that the inverted Chinese characters are processed more slowly than upright ones in both the orientation judgment task and the one-back identity matching task. Previous studies (e.g., Guan and Fang, 2002; Yeh et al., 2003) have demonstrated that performance in Chinese character processing is affected by the age at which the characters were learned, and this may be the reason why the inversion effect was observed stably, both in accuracy and response time analysis, in our study. The stimuli used in our study were learned in the first grade of primary school. We aimed to design research to test this hypothesis in the future.

### The developmental trajectory of the left-side bias effect in Chinese character processing

The left-side bias effect, another behavioral marker of expert processing, refers to the process by which a chimeric face created from the left-side of a face (from the viewer’s perspective) and its mirror image are considered more similar to the original face than a chimeric face created from the right side of the same face and its mirror image (Brady et al., 2005; Li and Cao, 2017). A left-side bias has also been observed for non-face stimuli, such as Chinese characters (e.g., Hsiao and Cottrell, 2009; Tso et al., 2014; Chung et al., 2017). The results of this study showed that left-side bias in Chinese character processing was stable in adults, which is consistent with previous studies (e.g., Hsiao and Cottrell, 2009; Tso et al., 2014; Chung et al., 2017; Li and Cao, 2017). Hsiao and Cottrell (2009) demonstrated a clear left-side bias in healthy adult readers of Chinese with mirror-symmetric Chinese characters as the stimuli. Tso et al. (2014) also found that writers with expertise and those of more limited writing abilities both showed a stable bias toward the left side. Importantly, in this study, left-side bias in Chinese character processing was not observed in the first-grade students, which suggests that 1-year experience of learning Chinese characters in a normal school environment is not enough learning experience in which to develop a left-side bias. Interestingly, in the third-grade students, a stable left-side bias was observed, and the bias effect was similar to that of adults. It suggested that with about 3-year learning experience, a stable left-side bias in Chinese character processing can occur. The previous studies demonstrated that both reading habits and the maturation of the neural mechanisms (e.g., visual word form area) necessary for expertly processing Chinese characters may contribute to a left-side bias (Li and Cao, 2017). Our future research will reveal what roles reading and neural mechanisms play in the developmental trajectory of left-side bias in processing Chinese characters. Moreover, previous event-related potential studies demonstrated that electrophysiology markers related to perceptual expertise of Chinese characters (e.g., the left lateralization of the N170) have occurred in Chinese students by second grade (Cao et al., 2011). Future studies will be designed to reveal whether the left-side bias of Chinese character processing can also be observed in second-grade students.

### The difference in the developmental trajectory between the inversion effect and the left-side bias effect in Chinese character processing

Although both the inversion effect and left-side bias in Chinese character processing develop with age, there are many differences in their developmental trajectories. The first difference is that the inversion effect was observed in the first-grade students, but left-side bias was first observed in the third-grade group. Our data support that the development of configural processing (measured by the inversion effect) occurs earlier than the development of left-side bias processing (measured by the left-side bias effect). These differences may be due to many factors. The most important factor may be the visual experience of the stimuli: mirror-symmetric Chinese characters are a small proportion of the total Chinese characters, so the students have much more visual experience of normal upright characters than mirror-symmetric ones. Consequently, the inversion effect in character processing emerges earlier than the left-side bias effect. The second difference in the developmental trajectories is that in terms of left-side bias, the third-grade students were similar to the adults; however, for the overall performance, the similarity to the adult group was not observed until the fifth grade. Interestingly, although left-side bias emerges later than the inversion effect, it matures earlier than the inversion effect; namely, the left-side bias effect can develop to the same level as an adult’s with less than 3 years of experience in Chinese character processing, whereas the inversion effect of Chinese character processing needs about 5 years of learning experience to reach maturity. The results suggested that expert perception processing of Chinese characters may develop more slowly than left-side bias processing. Future studies will be designed to test the hypothesis with other categories of expert stimuli.

Comparing the maturation time of left-side bias and the inversion effect between Chinese character processing and face processing may reveal many interesting hypotheses that can contribute to our understanding of the formation of expertise in human beings. For the inversion effect, previous studies have demonstrated that it takes many years for expert perception processing for faces to mature. For example, most studies have found inversion effects in children from 4 to 7 years of age (Young and Bion, 1980; Flin, 1985; Carey and Diamond, 1994; Tanaka et al., 1998; Brace et al., 2001; Pascalis et al., 2001; Mondloch et al., 2002; Pellicano and Rhodes, 2003), but expert perception processing for faces appears to mature, in the majority of children, between 10 and 13 years of age (Mondloch et al., 2002; Taylor et al., 2004). Interestingly, this study demonstrated that expert perception processing for Chinese characters matures at 10–12 years of age, which is similar to the age of face expert perceptual maturation. This raises an interesting question as to whether, with long-term experience, all expert perception processing may mature at this age, in accordance with the developing maturity level of the brain. With regard to the left-side bias effect, previous face processing studies have found that the maturation age is about 9–10 years, although there were some differences in the different tasks. For example, 9–10 years of age was shown to be the maturity age regarding the identity/gender task (Balas and Moulson, 2011; Proietti et al., 2015), but in the emotional task, it was around 7.5–12 years of age (Levine and Levy, 1986; Failla et al., 2003; Aljuhanay et al., 2010). In this study, the third-grade students (8–9 years old) have a similar left-side bias effect in Chinese character processing to that of adults. These results suggest that there is similar maturation age for left-side bias between different objects of expertise. Together, it may suggest that there is a similar maturation age for different expert stimuli processing related to both the inversion effect and the left-side bias effect.

## Conclusion

Consistent with previous studies, this study found a significant inversion effect and left-side bias effect in Chinese character processing in adults; more importantly, the inversion effect emerged by Grade 1, and the left-side bias effect did not emerge in the Grade 1 students but reached the same level as the adult group by Grade 3. It revealed that different expertise markers in Chinese character processing have different developmental trajectories.

## Data availability statement

The raw data supporting the conclusions of this article will be made available by the authors, without undue reservation.

## Ethics statement

The studies involving human participants were reviewed and approved by the ethical approval was obtained from the Zhejiang Normal University Ethics Committee. Written informed consent to participate in this study was provided by the participants’ legal guardian/next of kin.
